# Narratives unveil knowledge and awareness-related issue, reinforcing patients’ self-identity in sickle cell disease

**DOI:** 10.1186/s13023-026-04212-w

**Published:** 2026-02-19

**Authors:** Lucia De Franceschi, Carla Galvani, Giovan Battista Ruffo, Raffaella Colombatti, Giovanna Graziadei, Giovanni Palazzi, Donatella Venturelli, Valeria Maria Pinto, Alessandra Quota, Annalisa Scopinaro, Raffaele Vindigni, Paola Chesi, Gian Luca Forni, Antonia Gigante, Antonietta Cappuccio, Alessandra Fiorencis, Francesca Begali, Jacopo Ceolan, Silvia Vitale, Simone Villaboni, Maria Giulia Marini

**Affiliations:** 1 Department of Engineering for Innovative Medicine, University of Verona, Policlinico GB Rossi, AOUI, Verona, Italy; 2 THADREV Onlus, Verona, Italy; 3 U.O. Ematologia con Talassemia, ARNAS Civico Di Cristina Benfratelli, Palermo, Italy; 4 Clinic of Pediatric Hematology Oncology, Department of Child and Maternal Health, Azienda Ospedaliera University of Padova, Padova, Italy; 5 Department of Medical Area, Unit of Medicine and Metabolic Disease, Fondazione IRCCS Ca’ Granda Ospedale Maggiore Policlinico, Milan, Italy; 6 Dipartimento Integrato Materno Infantile U. O. Complessa di Pediatria Università degli Studi di Modena e Reggio Emilia, Modena, Italy; 7 Department of Transfusion Medicine, University Hospital of Modena, Modena, Italy; 8 Ematologia e Terapie Cellulari, IRCCS Ospedale Policlinico San Martino, Genova, Italy; 9 U.O.S Talassemia PO Vittorio Emanuele, Gela, Italy; 10 UNIAMO Federazione Italiana Malattie Rare APS ETS, Roma, Italy; 11 UNITED Federazione Nazionale delle Associazioni Talassemia, Drepanocitosi e Anemia Rare, Ferrara, Italy; 12 Healthcare Area, ISTUD Srl, Milano, Italy; 13 For Anemia Foundation, Genova, Italy

**Keywords:** Sickle cell disease, Narrative medicine, Narratives, Quality of life

## Abstract

**Background:**

Sickle cell disease (SCD) increasingly requires a holistic approach. Narratives have been used to complement clinical and observational findings related to quality of life (QoL). The “Sickle Cell Anemia Narrations” project aimed to help frame the illness burden and QoL of the multi-ethnic population with SCD in Italy via a narrative approach.

**Results:**

Twenty-one adult patients with SCD and 10 informal caregivers from seven SCD centers and two Patient Associations volunteered for this project. Researchers collected anonymous narratives and independently analyzed them through content analysis. Lack of SCD knowledge on multiple levels was found to strongly impact the illness experience independent of ethnicity. Fear of stigma at school, the challenging management of vaso-occlusive crises, and a lack of SCD knowledge at the workplace were reported. Fifty-five percent of participants reported a lack of SCD knowledge among healthcare professionals working outside the expert centers and 33% reported misdiagnosis. Caregivers highlighted the lack of coordination among territorial healthcare facilities.

**Conclusions:**

Our findings confirm that SCD represents a critical burden for patients and caregivers and indicate that lack of SCD knowledge may bolster the barriers to care, thus revealing the urgent need to enhance awareness to foster inclusion and quality of care.

## Letter to the editor

Sickle cell disease (SCD) is a rare red cell disorder due to a point mutation in the gene encoding the β-globin chain of hemoglobin (Hb), resulting in the production of pathologic (HbS) [1]. The main clinical manifestations of SCD include chronic hemolytic anemia and acute sickle cell-related vaso-occlusive crises (VOCs) responsible for acute and chronic organ damage [2, 3]. This negatively impacts patients’ survival rate and quality of life (QoL) [4, 5], reducing patients’ engagement in recreational activities, satisfaction with life, social interaction, and performance [6–9]. Indeed, a survey of 115 patients with SCD reported an employment rate of 39% compared to healthy population, which becomes more worrisome for women with SCD being 3 times more likely to be unemployed when compared to men with SCD. [6, 10] This implies a significant caregiver burden, requiring extensive family involvement for daily patient management [11–14]. Up to 30% of people with SCD are estimated to suffer from depression [15], and the priority treatment goal stated by patients in a recent international survey is QoL improvement [7].

A narrative approach has been used in the context of rare diseases and other therapeutic areas to identify key elements to improve the quality of care [16, 17]. Previous studies have shown that narratives complement QoL-related clinical and observational findings, helping to refine investigation tools, to provide new insights into the illness experience and to improve the clinical management of patients [18, 19]. In Europe, national programs on rare diseases have been developed to support both clinician and patients with rare diseases such as SCD. In Italy, the national center of rare diseases (CNMR) has highlighted the role of narrative medicine as key tool to support patient’s empowerment and to reinforce patients’ self-identity. [4, 20]

In Italy, the population of patients with SCD presents a multi-ethnic background, including endemic patients of Caucasian origin and first- or second-generation patients of African descent [21]. Thus, Italy represents a unique setting to examine the experience of patients with SCD. Here, we report the results of the “Sickle Cell Anemia Narrations” (SCAN) project, aimed at framing the burden of illness and QoL of the multi-ethnic population with SCD in Italy using a narrative approach. The project was developed as part of the DB-INTHEM initiative—the Database for the Italian Network of Thalassemia and Hemoglobinopathies.

SCAN was conducted in accordance with the Declaration of Helsinki and ICH guidelines for Good Clinical Practice. Ethical approval was obtained (document no. 264/2019). The present study was carried out between January and September 2021. Adult patients with SCD and their parental or spousal caregivers were the targets of this study. Twenty-one patients with SCD and 10 caregivers from seven Italian centers for SCD and two Patient Associations volunteered. Age ≥ 18 at recruitment, a confirmed SCD diagnosis irrespective of the phenotype, or the informal caregiving of a patient with SCD constituted the eligibility criteria for participants; however, the ability to write or communicate in Italian was critical. Participants were asked to join the project by entering the Alchemer platform, available on the project webpage http://medicinanarrativa.eu/scan and aimed to collect participants’ narratives and data anonymously. Participants were asked to answer to a survey addressed to collect sociodemographic (Table 1) and to write their narrative following an illness plot, characterized by evocative narrative inputs [22] with a chronological flow to identify the illness experience’s evolutions over time [23]. This strategy brings out critical qualitative experience of the disease and integrates the perspectives of patients-caregivers-health care providers, overcoming the limitations of QoL questionnaires [24–26]. Before accessing the investigation tools, participants completed an online informed consent form after being briefed on the research purposes and European confidential data processing procedures [27]. Survey data were analyzed through descriptive statistics. Researchers entered anonymous narratives into the Nvivo software for coding [28]. Three narratives for each group were collectively coded to assess consistency across team members and then reviewed during weekly peer debriefings to reach a shared data understanding. Researchers employed open interpretive coding to analyze the identified topics.

**Table 1:** Sociodemographic data of participants

	Patients (N = 21)	Caregivers (N = 11)
**Gender**		
Women	13 (62%)	8 (73%)
Men	8 (38%)	3 (27%)
**Age, average** ± **SD**	33 ± 8	46 ± 10
**Ethnic background**		
Caucasian	11 (52%)	8 (73%)
African	7 (33%)	3 (27%)
Mixed Caucasian-African	3 (14%)	-
**Region of residence**		
Northern Italy	11 (52%)	7 (64%)
Central Italy	1 (5%)	-
Southern Italy	7 (33%)	4 (36%)
Other country	1 (5%)	-
Non-responders	1 (5%)	-
**Education**		
Intermediate school	3 (14%)	-
High school	9 (43%)	9 (82%)
Bachelor/Master	9 (43%)	2 (18%)
**Civil status**		
Single	6 (21%)	1 (9%)
Married/Cohabitant	14 (67%)	9 (82%)
Separated	1 (5%)	1 (9%)
**Employment status**		
Student	2 (10%)	1 (9%)
Working	10 (48%)	9 (82%)
Unemployed/Not working	5 (24%)	1 (82%)
Retired	3 (14%)	-
Non-responders	1 (5%)	-

Data are presented as n (%) or average ± standard deviation (SD)

Weakness and pain negatively impacted QoL in the narratives of 53% and 41% of patients with SCD, respectively. Sixty-three percent of patients reported losing an average of 50 ± 32 working days annually due to SCD, while 41% reported losing their jobs due to acute pain episodes and frequent hospitalization (Table 2). Fear of stigma during school years was spontaneously reported; nonetheless, being successful in academics or work, and openly talking about SCD was experienced as redemption. Management of challenging VOCs and lack of SCD knowledge at the workplace were other topics identified from patients’ narratives. Forty-four percent of patients described the care pathway as initially complex but currently positive, indicating a relationship of trust between patients, caregivers, and healthcare professionals; in comparison, 28% explicitly underlined that this had become satisfactory when they found the expert center after visiting several healthcare facilities. Thirty-three percent of patients and 17% of narratives referred to Transitional Care. Fifty-six percent of participants reported a lack of SCD knowledge among healthcare professionals outside the expert centers. Overall, caregivers pointed out that the diagnosis was communicated with poor empathy and that the referral centers, general practitioners, and local healthcare facilities lacked coordination, which increased the caregiving burden. Forty-eight percent of patients and 50% of caregivers considered the writing experience a source of well-being, while 32% of patients and 13% of caregivers defined it as liberatory. The narratives of SCD patients from different ethnicities did not differ crucially.

**Table 2:** Participants’ experience of SCD at the workplace, school, and throughout the care pathway: quotes from narratives

	Experiencing SCD at the workplace/at school	Experiencing SCD throughout the care pathway
**Patients**	*–My schoolmates were not supposed to know that I was ill; when they perceived something, I pushed them away. I didn’t want them to find out about my different condition. Only two of my friends knew about my illness. This was also so that they could help me when crises happened.* (Patient 005) *–At school and at work, I was always the big absentee: long periods of absence, which I then had to make up for. At school, I missed a lot of lessons, but I always managed to do well: a few debts in high school, but never a failure. At university, a triumph.* (Patient 018) *–My health does not guarantee continuity in work. When I have crises, that’s when I feel the most difficult. If I have it at night, I am destroyed in the morning. In the past, I was also left home, on leave, not really knowing what that meant. I waited, and nothing happened, they didn’t call me, so I realized they didn’t want me anymore. […] I have tried to explain my illness, but sometimes I feel they don’t understand.* (Patient 009)	*–They were not up to the task. They did not know the disease, did not understand that it was sickle cell anemia, and said it was microcytosis.* (Patient 004) *–Before, doctors were annoying; now, they are an essential reference point because of their preparation. The treatments used to be a burden; now, I perceive them as improving my lifestyle.* (Patient 007) *–It was sad to hear that I had a genetic disease that meant I would not be able to recover and even that I risked passing it on to my eventual children. It is devastating to hear these things. When I heard that I might lose my eyes, I lost the will to fight for life.* (Patient 002)
**Caregivers**	*–The illness did not prevent him from leading an active life (despite the absences); he attended school well. In the summer, he was a cook’s helper. Everything was fine until the virus and the consequences of the necrosis of the femoral head.* (Caregiver 007) *–Even at school, there was some difficulty in following and understanding things. She often stayed home from school; one week, she was in the hospital for a month and could not follow the programs.* (Caregiver 009) *–Especially during her teenage years, she missed many days of school due to transfusions and various hospitalizations; she flunked out for two years, marking her entire course of study. Despite myriad difficulties, she completed her three-year degree, taking a little longer, but she did it!* (Caregiver 010)	*–The professionals in the department mistreated us. They took a blood sample and told us it would be at least twenty days before the response but that my daughter was ill anyway. Stop. Without explaining anything to us, they sent us home.* (Caregiver 008) *–Luckily there is a specialized center not far from us. In the rest of Italy, the situation is not like that, although we know more about this disease. […] There was no connection between the family doctor and the specialists. Sometimes I felt that I was the go-between among the health professionals. The health network is not yet prepared to receive family members’ experiences and systematically communicate with each other. I would not like psychological support to be understood only as being addressed by the psychologist or psychiatrist who meets the patient or family once a month, perhaps not knowing the illness. There is not only the illness but the experience of disease, which is a human aspect that should be implemented.* (Caregiver 007) *–The professionals who should have considered my child’s deafness were not as concerned as I expected. The child no longer spoke. Before the infection, she had started with the first little words, then silence.* (Caregiver 002)

Taken together, our findings confirm that SCD represents a critical burden for patients and their caregivers [7]. Participants’ narratives indicate that lack of SCD knowledge strongly impacts their illness experience on multiple levels: during school years and at the workplace, where managing VOCs remains challenging [5] and may lead to perceived stigma [29], and throughout the care pathway, from first symptoms to current management. Lack of SCD knowledge is a global problem [30–32], and its resolution at various stages could foster patient inclusion and minimize fear [7], along with therapeutic progress in pain management. This agrees with a recent study exploring the experience of living with SCD and sickle cell related pain in adolescent and young adults referring to SCD comprehensive centers in the United States [33]. The care experience and the stigmatization present in the narration of these patients, displaying a lot of similarity with our patients’ narrative data, suggesting that the experience of living with SCD might be transversal to different cultures. Thus, reflective writing as a source of PROs may boost patients’ SCD awareness, self-identity [4], and their relationships with caregivers and healthcare professionals (HCPs). Its contribution toward implementing personalized SCD illness education may also be explored. Our narrative data also suggest that lack of SCD knowledge may bolster the barriers to care, already reinforced by the poor integration among expert centers, territorial medicine, and regional differences in the Italian National Healthcare System [34]. Indeed, the intersection of patients’ narrative data and physicians involved in care of SCD patients has been reported to improve the communication and the negotiation phase of HCPs by reducing the stereotypes on patients with SCD [35–39]

Thus, increasing the dialogue between referral centers and territorial medicine to guarantee continuity and implementing integrated clinical pathways to support patients’ transition and special situations, such as pregnancy, remain critical. Moreover, a homogeneous territorial presence of expert centers for SCD may be strategically important to reduce misdiagnosis, inaccurate interventions, and emergency room admissions. This will contribute to ensure the optimal clinical management of patients with rare disease, to improve treatment adherence and to develop coping strategies. All of this contributes to the improvement of patients’ QoL.

The SCAN project suffers from some limitations, such as the voluntary nature of patient participation, the involvement of patients who wanted to narrate their own experience of living with SCD, and the inclusion of SCD patients already effectively followed up by comprehensive centers for hemoglobinopathies.

## Conclusions

**Fig. 1:**
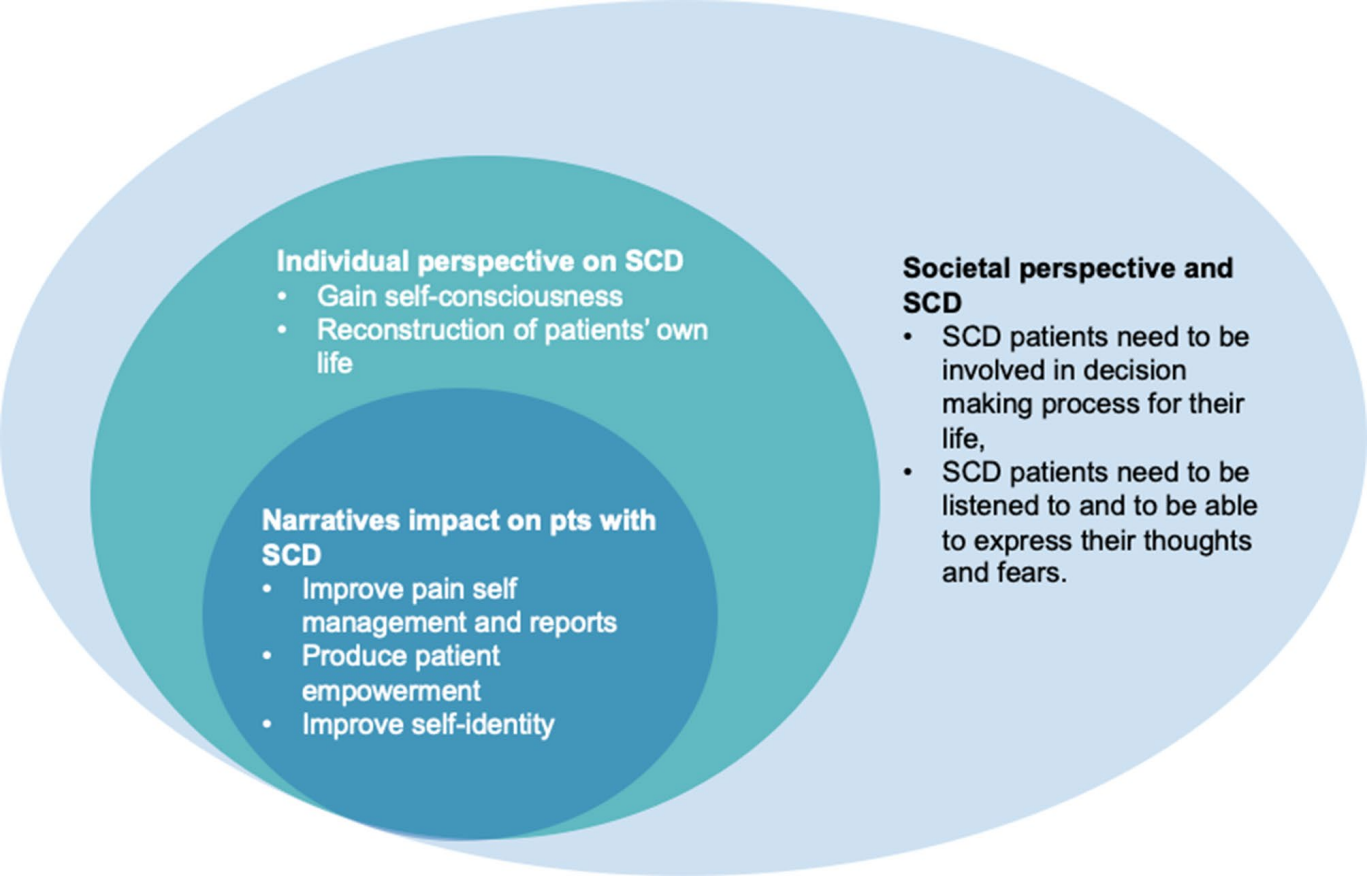
Fostering sickle cell disease (SCD) awareness at multiple levels through a narrative approach. The clinical management of patients with sickle cell disease should take account of societal and individual perspective in designing a comprehensive health program for sickle cell disease (SCD). Patients’ narratives are a central tool to reinforce self-identity and to empowerment of patients. This will positively impact both individual perspective of SCD and the ability of patients to deal with symptoms related to SCD. Pts: patients, SCD: sickle cell disease

Our findings aim to advance the understanding of the personal, social, and daily management aspects of SCD, which is still weighted down by health-related stigma and marginalization [40, 41]. Indeed, our study highlights that the experience of living with SCD is transversal to different cultures and how patients’ narratives might integrate the individual and societal perspective on SCD (Fig. 1). SCAN represents the first application of the narrative approach in SCD. We believe this approach could be critical in fostering SCD awareness on multiple levels (Fig. 1). The gained insights may inform future studies to implement SCD knowledge at an individual and societal level while better equipping the territorial SCD healthcare network to improve patients’ QoL.

## Data Availability

All datasets used and analyzed during the current research are available in Italian from the corresponding author, upon reasonable request.

## Abbreviations

SCD: Sickle Cell Disease
VOC: vaso-occlusive crises
ICH: International Council for Harmonization of Technical Requirements for Pharmaceuticals for Human Use
QoL: quality of life
HCPs: Healthcare Professionals.
